# Termination of Palliative Chemotherapy Near the End of Life: A Retrospective Study of Gastrointestinal Cancer Patients

**DOI:** 10.1089/pmr.2023.0027

**Published:** 2023-07-19

**Authors:** Yoshifumi Matsumoto, Akito Higuchi, Marika Shiba, Kenta Sasaki, Takuro Saiki, Yujiro Honma, Kazuyoshi Kimura, Qiliang Zhou, Yasuo Saijo

**Affiliations:** ^1^ Department of Medical Oncology, Niigata University Graduate School of Medical and Dental Sciences, Niigata, Japan.; ^2^ Palliative Care Team, Niigata University Medical and Dental Hospital, Niigata, Japan.; ^3^ School of Medicine, Niigata University, Niigata, Japan.

**Keywords:** chemotherapy, end of life, gastrointestinal cancer, termination

## Abstract

**Background:**

Palliative chemotherapy is commonly used for advanced cancer patients. The timing of chemotherapy termination is crucial for efforts to maintain quality of life.

**Patients and Methods:**

This retrospective study included gastrointestinal cancer patients who were treated with chemotherapy and died between 2013 and 2022 at Niigata University Medical and Dental Hospital. Data were reviewed regarding age, gender, cancer type, reason for chemotherapy termination, cause of death, survival after chemotherapy termination, and place of death.

**Results:**

In total, 388 patients were included; the median survival after chemotherapy was 73 days. Patients aged <67 years had shorter survival durations (59 days), compared with patients aged >67 years (82 days). Ten (2.6%) patients began a new chemotherapy regimen, whereas 17 (4.4%) patients received chemotherapy, within 4 weeks before death. The most common reason for chemotherapy termination was disease progression, and most deaths occurred in hospitals.

**Conclusion:**

The rates of chemotherapy and initiation of new chemotherapeutic regimens near the end of life were lower than previously reported. Most deaths occurred in hospitals, highlighting the need for development of hospices.

## Background

Advanced cancer patients are often treated with palliative chemotherapy, which improves symptoms and quality of life, while prolonging survival in metastatic cancers.^1^ Although advances in palliative chemotherapy have prolonged the overall survival of advanced cancer patients, eventual mortality is inevitable. Accordingly, these patients are often treated with aggressive chemotherapy, even near the end of life. Chemotherapy near the end of life can cause severe adverse effects, thereby increasing use of the emergency department and the rate of emergent admissions.^2,3^

Aggressive treatment also imposes a burden on the family members and health care professionals, and it increases the financial cost. Furthermore, it impairs quality of life because of the adverse effects of chemotherapy and the unnecessary use of intensive care treatment. Palliative chemotherapy does not improve the near-death quality of life (QOD) in patients with a moderate or poor performance status, and it worsens the QOD in patients with a good performance status.^4^ Chemotherapy termination is among the top five practices for improving patient care and reducing medical costs.^5^ Although the decision to end treatment is challenging for both physicians and patients, there is a need to avoid unnecessary harm to the patient.^6^

Several retrospective studies have revealed a 7%–52% frequency of palliative chemotherapy in the final month among near-death patients.^2,7–9^ This frequency may vary according to cancer site, health care system, and culture.

In this study, we retrospectively collected data regarding metastatic gastrointestinal cancer patients near the end of life, all of whom received palliative chemotherapy at our institution. We determined the survival rate after chemotherapy termination, as well as factors influencing this decision and factors influencing death in this patient population.

## Methods

### Data collection

This study was performed in accordance with the Declaration of Helsinki. The study protocol was approved by the ethics review committee of the School of Medicine of Niigata University (approval no.: 2022-0383). This was a retrospective observational study, and it did not involve any clinical interventions. Consent to participate was obtained through the opt-out method on the School of Medicine Niigata University website. Bereaved family members of the patients were allowed to opt out of the study through the Internet homepage of Niigata University School of Medicine.

Data were retrospectively collected for gastrointestinal cancer patients treated with palliative chemotherapy at the Outpatient Chemotherapy Center of Niigata University Medical and Dental Hospital, with dates of death between January 2013 and September 2022. The data included age, gender, cancer type, number of chemotherapy regimens, reason for chemotherapy termination, time between the last treatment and death, cause of death, and place of death. Chemotherapy included cytotoxic drugs, molecular targeting drugs, and immune checkpoint inhibitors.

### Statistical analyses

The chi-squared test and Fisher's exact test were used to analyze differences between groups. Survival duration was defined as the time between the last chemotherapy session and death from any cause. Survival curves were drawn using the Kaplan–Meier method and compared using the log-rank test. *p*-Values <0.05 were considered statistically significant. Statistical analyses were performed using IBM SPSS Statistics, ver. 22.0 (IBM Corp., Armonk, NY, USA).

## Results

### Patient characteristics

Data were extracted from the electrical medical records of 388 patients who had been treated with palliative chemotherapy and had confirmed dates of death (Table 1). The median age was 67 (range, 21–91) years. There were 246 men and 142 women. The patient population included colorectal (*n* = 124), pancreatic (*n* = 97), bile duct (*n* = 61), stomach (*n* = 59), and esophageal (*n* = 47) cancer patients.

**Table 1.** Patient's Characteristics (*N* = 388)

Characteristics	No.	%
Age (years old)	67 (21–91)	
Gender		
Male	246	63.4
Female	142	36.6
Cancer site		
Esophagus	47	12.1
Stomach	59	15.2
Colorectal	124	32.0
Pancreas	97	25.0
Bile duct	61	15.7
Chemotherapy regimen		
1	109	28.1
2	138	35.6
≥3	141	36.3
New chemotherapy regimen		
<2 Weeks	0	0.0
2–4 Weeks	10	2.6
4–12 Weeks	68	17.5
≥12 Weeks	320	79.9
Time to death from last chemotherapy		
<2 Weeks	17	4.4
2–4 Weeks	24	6.2
4–12 Weeks	169	43.6
≥12 Weeks	178	45.9

### Chemotherapy and reasons for termination

One, two, and three or more chemotherapy regimens were administered to 109, 138, and 146 patients, respectively (Table 1). The representative two regimens of each type of cancer were cisplatin +5FU and nivolumab in esophagus, oxaliplatin+S-1 and paclitaxel+ramucirumab in stomach, FOLFOX±bevacizmab and FOLFIRI±bevacizumab in colorectal, FOLFIRINOX and nab-paclitaxel+gemcitabine in pancreas, and cisplatin+gemcitabine and S-1 in bile duct. Ten (2.6%) patients had begun a new chemotherapy regimen within 4 weeks, whereas 68 had begun treatment between 4 and 12 weeks, before death. The survival durations were <2, 2–4, 4–12, and >12 weeks in 17 (4.4%), 24 (6.2%), 169 (43.6%), and 178 (45.9%) patients, respectively.

The reasons for chemotherapy termination are summarized in Table 2. Disease progression was the most common reason, followed by adverse events and severe complications leading to poor health or death. Forty-three cases of adverse events contained poorer performance status (11 cases), infection (11 cases), interstitial lung disease (10 cases), skin disorders (2 cases), bone marrow suppression (2 cases), neuropathy (2 cases), diarrhea (2 cases), and others (3 cases). Sudden death occurred in seven patients. Among the 17 patients treated within 2 weeks before death, adverse effects, severe complications, and sudden death occurred in 1, 13, and 3 patients, respectively; no patients experienced disease progression.

**Table 2.** Reason of Chemotherapy Termination

	Chemotherapy in 2 weeks	Chemotherapy in 4 weeks	Chemotherapy in 12 weeks	All cases (include over 12 weeks)
Total	17	41	210	388
DP	0	18	151	307
AE	1	3	26	43
SC	13	19	26	30
Will	0	0	0	1
SD	3	3	7	7

AEs, adverse effects; DP, disease progression; SC, severe complication; SD, sudden death.

### Survival after chemotherapy termination

**FIG. 1.**
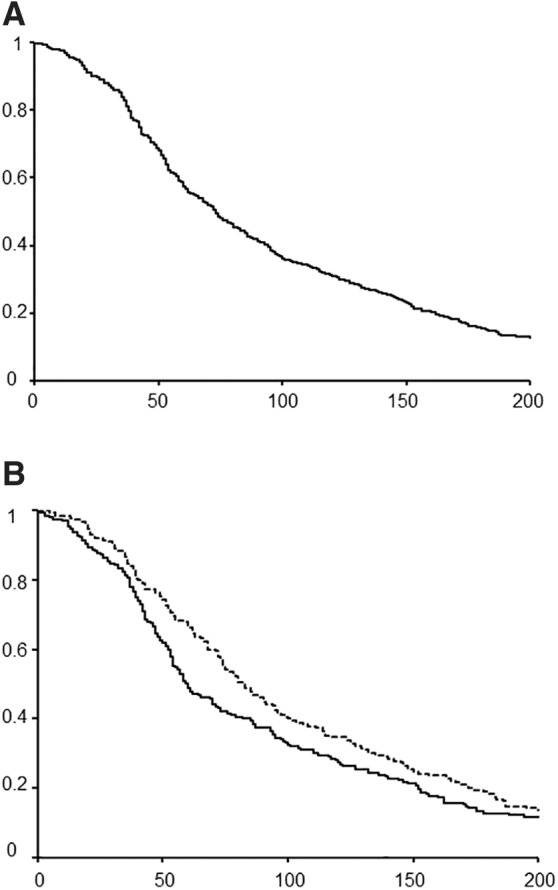
**(A)** Overall survival after chemotherapy termination. **(B)** Survival rates in patients aged <67 and ≥67 years.

The overall survival curve after chemotherapy termination is shown in Figure 1A. The median survival time was 73 days. Because median age in this study was 67 years old, we divide the patients into two groups of younger and older by 67. The median survival time was shorter for patients aged ≤67 years (59 days) than for patients aged >67 years (82 days), but the difference was not statistically significant (*p* = 0.097) (Fig. 1B). There were no survival differences according to the number of chemotherapy regimens, cancer site, or gender.

The causes of death according to survival duration are summarized in Table 3. As expected, disease progression was the most common cause of death, followed by infection and bleeding. In contrast, bleeding and sudden death were more common among patients treated within two weeks before death.

**Table 3.** Cause of Death

	Chemotherapy in 2 weeks	Chemotherapy in 4 weeks	Chemotherapy in 12 weeks	All cases (include over 12 weeks)
Total	17	41	210	388
DP	7	19	168	332
Bleeding	3	6	13	14
Infection	1	7	13	18
TE	1	2	8	8
AE	0	2	2	2
SD	3	5	6	14

TE, thromboembolism.

### Place of death

Most patients died at a hospital, followed by hospices and their homes (Table 4). A greater proportion of patients with survival durations <2 weeks died at our hospital. The proportions of deaths at other hospitals and hospices were greater among patients with longer survival durations (20.0% within 4 weeks vs. 53.9% over 4 weeks).

**Table 4.** Location of Death

	Chemotherapy in 2 weeks	Chemotherapy in 4 weeks	Chemotherapy in 12 weeks	All cases (include over 12 weeks)
Total	17	41	210	388
Our hospital	12	33	130	193
Other hospitals	3	5	57	131
Home	2	3	13	26
Hospice	0	0	10	38

## Discussion

This study retrospectively investigated the outcomes in gastrointestinal cancer patients after termination of palliative chemotherapy. The median survival after chemotherapy termination was 71 days, and older patients tended to survive longer than younger patients (82 days vs. 59 days). Seventeen (4.4%) and 41 (10.6%) of the 388 patients died within 2–4 weeks of the last treatment. Ten (2.6%) patients began a new chemotherapy regimen in their last month of life. Although half of the patients died of disease progression, the proportions of bleeding, infection, and sudden death were greater in patients with a survival duration of ≤4 weeks. Although most deaths occurred in hospitals, the proportions of home and hospice deaths were greater among the patients with longer survival durations.

Chemotherapeutic advances, including molecular targeting drugs and immune checkpoint inhibitors, improve the survival and quality of life in patients with advanced or recurrent cancers.^1^ However, the cure rates for advanced solid cancers are quite low, and mortality is inevitable. Chemotherapy increases the health care cost and can cause significant adverse effects, reducing quality of life.^5^ Therefore, chemotherapy termination may be crucial for cancer patients near the end of life. However, the decision for chemotherapy termination is challenging for both health care professionals and patients.^6^

Earle et al. identified the indicators of quality of cancer care using Medicare claims of 48,906 cancer patients who died between 1991 and 1997.^10^ The five quality indicators included (1) <10% of patients received chemotherapy in the last 14 days; (2) <2% of patients began a new chemotherapy regimen in the last 30 days; (3) <4% of patients had emergency department visits, intensive care unit admissions, or multiple hospitalizations in the last 30 days; (4) <17% of patients died in acute care institutions; and (5) ≥55% of patients received hospice services before death, and <8% of these patients were admitted to hospice within the last 3 days.

Although chemotherapy has dramatically improved since the publication of the study by Earle et al., these indicators may continue to be used for cancer patients near the end of life. A prospective observational cohort study of patients with end-stage cancer (median survival after chemotherapy: 3.8 months) revealed worse QOD in patients with a good performance status and no improvements in QOD among patients with poor performance status, compared with patients who did not receive chemotherapy.^4^

Despite evidence in multiple studies,^4,5,10^ the number of patients near the end of life who receive chemotherapy has not decreased. It has been reported that >10% of patients with breast,^11,12^ gastrointestinal,^8,13^ and other^2,3,9,14^ cancers receive palliative chemotherapy during the last four weeks of their lives. Several studies have identified younger age as a predictor for chemotherapy use during the last months of life.^8,11–14^ Other reported predictors include albumin level,^11^ socioeconomic status,^9^ presence of caregivers,^14^ cancer type,^7^ and performance status.^15^

Only one small study has investigated the use of chemotherapy near the end of life in Japan.^16^ Chemotherapy was performed in 32% of patients with solid tumors during the last month of life, with increased use of molecular targeting drugs and immune checkpoint inhibitors. In contrast, this study revealed lower rates of chemotherapy (4.4% and 10.6% in last 2 and 4 weeks, respectively), and only 2.6% of the patients began a new chemotherapy regimen during the last four weeks. Consistent with findings in previous studies, older patients tended to have longer survival durations after chemotherapy termination, compared with younger patients.

Longer survival of older patients denotes early termination of palliative chemotherapy compared with younger patients. There might be two reasons. First, the older patients often have poorer performance status and comorbidities, thus appropriately terminate chemotherapy. Second, the older patients simply do not wish to continue palliative chemotherapy near end of life. There were no other factors influencing the overuse of chemotherapy near the end of life.

Reasons for higher rates of chemotherapy near the end of life may be related to both patient and physician factors. Patients may prefer to continue chemotherapy and prolong their lives, even at the cost of reduced quality of life. They often are not concerned about adverse effects.^13^ The decision of chemotherapy termination is also challenging for physicians because the primary aim of cancer treatment is to prolong survival. In this study, nearly half of the patients died of disease progression, even among patients with survival durations <4 weeks, whereas the remaining patients died of cancer and treatment complications.

An accurate estimation of the expected survival duration may prevent the overuse of chemotherapy and reduce the rates of hospitalization for chemotherapy complications. Palliative prognostic indexes have been proposed for survival prediction in advanced cancer patients near the end of life, which may facilitate decision making for chemotherapy termination.^17,18^

In this study, 84% of patients died in hospitals, including our acute care hospital and other acute and chronic care hospitals, whereas only 10% died in hospices. A previous study of seven developed countries showed that 22.6%–52.1% of patients died in acute care hospitals.^19^ This discrepancy may be related to differences in health care systems between Japan and other countries. In Japan, 80% of patients with fatal diseases, including cancers, die in hospitals. There are few hospice facilities in Japan. A few patients, who are cared for by private clinics, die in their homes. The hospice and home health care systems in Japan require substantial improvement.

Our study had some limitations. First, it was a retrospective observational study conducted in a single institution, and the sample size was small. We did not analyze the effects of palliative care on chemotherapy termination. However, we assume that most patients received palliative care because these services are easily accessible at our hospital. Early palliative intervention is associated with less chemotherapy during the last four weeks, along with fewer hospitalizations and greater use of hospices.^20^ Therefore, early palliative intervention should be promoted for metastatic advanced cancer patients.

## Conclusions

The overuse of chemotherapy can be avoided in gastrointestinal cancer patients near the end of life. However, most of these patients die in hospitals, rather than in hospices or their homes. These findings indicate that the palliative care system for cancer patients near the end of life in Japan requires substantial improvement.

## Abbreviations Used

AEs: adverse effects
DP: disease progression
QOD: near-death quality of life
SC: severe complication
SD: sudden death
TE: thromboembolism
